# Caregiver delay in seeking healthcare during the acute phase of pediatric illness, Kigali, Rwanda

**DOI:** 10.11604/pamj.2018.30.160.15286

**Published:** 2018-06-22

**Authors:** Christian Umuhoza, Angelique Charlie Karambizi, Lisine Tuyisenge, Peter Cartledge

**Affiliations:** 1 University of Rwanda, College of Medicine and Health Sciences, Kigali, Rwanda; 2 University Teaching Hospital of Kigali, Department of Pediatrics, Kigali, Rwanda; 3 Yale University, Rwanda Human Resources for Health (HRH) Program, Department of Pediatrics, Kigali, Rwanda

**Keywords:** Healthcare seeking, patient acceptance of health care, care delay, child, preschool, pediatrics, caregivers

## Abstract

**Introduction:**

Delay in seeking healthcare contributes significantly to under-five mortality. Multiple socioeconomic and demographic factors have been reported as predictors of such delay. There is no published research in this area in Rwanda. Our aim was to describe the caregivers' delay in seeking healthcare during the acute phase of a childhood illness among under-five children admitted in a tertiary hospital, Rwanda.

**Methods:**

This was an analytical, descriptive cross-sectional study conducted at University Teaching Hospital of Kigali. Bivariate analysis and logistic multivariate regression were used to analyze factors associated with delayed care-seeking behavior, defined as seeking care after the first 48 hours of illness onset.

**Results:**

Among 275 admitted children under age five, care-seeking delay occurred in 35% (97/275) of cases. The most significant predictors of delay in seeking care were use of traditional healers (AOR = 14.87, 95% CI: 3.94-56.12), the recognition of illness as mild (AOR = 8.20, 95% CI: 4.08-16.47), use of un-prescribed medicine at home (AOR = 2.00, 95% CI: 1.01-3.91), use of special prayers provided by ministers of God before seeking healthcare (AOR = 6.42, 95% CI: 2.50, 16.48), and first consultation at public institutions (AOR = 4.00, 95% CI:1.54-10.39).

**Conclusion:**

Even though Rwanda has made tremendous achievements in strengthening the community-based health systems, delayed care-seeking is a reality. Health education and behavior change communication interventions are needed at the community level to address the factors that lead to delay in seeking healthcare.

## Introduction

The under-five mortality rate in Rwanda has decreased significantly from 152 per 1000 in 2005 to 50 per 1000 in 2015 [1]. The majority of those deaths occur within the first year of life, with an infant mortality rate at 32 per 1000 and neonatal mortality rate at 20 per 1000 [2]. Although improving the quality of care in health facilities can play a role in reducing child mortality and morbidity [3], that approach alone is not enough [4]. Other determinants influencing access to adequate health-care play a great role in the reduction in childhood mortality, especially in developing countries [5]. Access to health-care facilities has been shown to influence the time spent at home before seeking healthcare when a child is sick [6]. According to a World Health Organisation report, 70% of child deaths are related to poor or delayed care-seeking, and can be prevented by seeking healthcare earlier [7]. Two studies defined *″Delay in seeking healthcare″* as staying home for 48 hours or more before consulting any formal healthcare facility [8,9]. Delay in seeking healthcare has been shown in several regions to play an important role in under-five morbidity and mortality, however the factors will vary within different countries, settings and communities, therefore a better understanding for each of these is necessary in order to improve care. For example, factors such as geographic accessibility (distance from home to healthcare facility) [10], economic affordability of care [11], religious beliefs [12], use of un-prescribed medicine [13], educational level of the care giver [14], age and sex of the sick child [15], age of the caretaker [16], family size and composition [16], use of traditional healers [17] and biased recognition of the severity of symptoms [18] have all been found to be associated with delay in seeking healthcare and associated to increased morbidity and mortality among under-five years children worldwide [19]. In Rwanda, there is no published evidence reporting if these associations are found in our population. A better understanding of the associated determinants to health seeking delay in our specific setting can assist healthcare providers and policy makers in meeting the needs of this population and to provide higher quality, accessible, care that is more satisfying to our patients, whilst decreasing the morbidity and mortality in children under-five. This research project therefore aimed to answer the question *″How prevalent is delay in seeking healthcare, what are its predictors and its immediate outcome in term of morbidity and mortality among children, under 5-years-of-age, in Rwanda″*

## Methods

**Study design and site:** We undertook a descriptive, analytical, cross-sectional study. The study was conducted in the pediatrics Emergency Department (ED) at the University Teaching Hospital of Kigali (CHUK), a national referral (tertiary) hospital in Kigali, the capital of Rwanda.

**Outcomes:** The primary outcome measure was the prevalence of caregivers' delay in seeking healthcare during the acute phase of illness. Delay in healthcare seeking was defined as greater than 48 hours after onset of illness [8,9]. Based on our literature review, described above, the predictors that we considered in our study were: 1) socio-demographic predictors of the child; 2)socio-demographic predictors of the principal caretaker; 3) economical predictors such as wealth index; 4) geographical accessibility; 5) clinical or illness related predictors Wealth (socio-economic status) is measured in Rwanda using the formal ′Ubudehe′ system between 1 to 4 with category-1 population being extremely poor and category-4 being the wealthiest. It is a multifactor system determined with members of the population classified in their community. The secondary outcome focused on the immediate outcomes at the level of CHUK in term of morbidity and mortality in relationship to delayed care-seeking behavior and the time spent at the previous referring hospital. Immediate mortality was defined as death within 48 hours of presentation. Morbidity was assessed in terms of i. admission to PICU in the first 24h, ii. CPR and/or BMV in the first 24 h of admission

**Questionnaire:** The questionnaire employed was based on the standardized and validated Explanatory Model Interview Catalogue questionnaire that has been used in a survey done in Zanzibar,2008-2009 about under-five childhood illnesses [20]. That questionnaire was in the English language and was therefore for this study it was translated into Kinyarwanda, the single, unifying language of Rwanda, by the Principle Investigator. The translated questionnaire was piloted on 20 patients to ensure understanding of the questions.

**Measurement of delayed presentation:** Within the questionnaire parents were asked the day (date) and time of the onset of illness. The date and time of presentation was also included in the data collection. The time difference between these was therefore used to calculate time to presentation.

**Study participants:** All children under-five presenting to the pediatrics ED, CHUK, from the 1^st^ September 2015 to 29^th^ February 2016 were invited to enroll. Children with any of the following were excluded: symptoms lasting for more than two months, readmitted children previously included in this study and children presenting with trauma.

**Sample size:** Based on an estimated prevalence of 20% for delay in seeking health care [21], alpha of 0.05 and a power of 80%, we aimed to measure within 7% and the final sample size was 275 patients.

**Procedures at enrollment:** An interview using a pretested questionnaire was used to collect all the information required. Interviews were undertaken for all the participants with none of them ′self-completing′ the questionnaire. This was to ensure understanding of the questions and to not exclude participants with poor literacy. To minimize recall bias due to fatigue, data was not collected during the night (6pm to 6am). Once the caretaker consented to be included in the study, he or she was questioned on one occasion at a single point of time. In order to assess morbidity and mortality, we reviewed the medical records and charts at the time of discharge and we reported if the patient was still alive or had died, whilst identifying the date and time of death.

**Statistical analysis:** Epidata 3.2 was used to store data and Stata 13.0 for statistical analysis. Univariate analysis was done for all predictors considered as risk factors, including socio-demographic characteristics of the caretakers and children, economical, geographical accessibility and illness related characteristics. The normality of distribution was tested using the Shapiro-Wilk W-test. In bivariate analysis, the children taken outside the home after the first 48 hours (the delay) were compared with those who consulted in the first 48 hours of illness (non-delay). This was done with respect to socio-demographic characteristics of the child and the caretaker, the economic characteristics, the geographical accessibility and the illness related characteristics. Pearson Chi-squared tests were used for independent intergroup comparison and association between the outcomes which were delay to seek medical care and categorical predictors. If the frequency by cell was five or less, the chi-square test was replaced by Fisher's exact test. The variables having a p-value of <0.15 in bivariate analysis were included in a multiple binary logistic model in order to identify the ′significant predictors′ for delay. We controlled for confounding by measuring the known confounders (sex and age) and including them as covariates in multivariate regression analysis.

**Ethics:** Caretakers were given an explanation of the study and signed a consent form written in Kinyarwanda. Risks to participants (physical, emotional, social, economic, legal) were all considered and found to be minimal. Participants were not given any incentives or payments for taking part in the study. This research proposal was submitted and approved by Institutional Review Board (IRB) of the Faculty of Medicine at the National University of Rwanda (Reference: CMHS/IRB/307/2015). This was followed by local review and approval at the research ethics committee of CHUK.

## Results

In total, 275 caregivers meeting our inclusion criteria consented to be included in the study. No caregivers declined to be involved in the study (100% recruitment rate). 120 children did not meet the inclusion criteria, and were therefore excluded. 178 caregivers (64.7%) sought care at the level of a qualified health facility in the first 48 hours of illness (Table 1).

**Table 1:** Demographic characteristics of all participants, CHUK (Children N=275, Caregivers N=275)

Characteristics	Total study population number	
	Frequencies	%
**Child's characteristics**		
Mean age of child	297 days (IQR: 94-777)	
Female sex	108	39.3%
**Caretaker's characteristics**		
Mean age of the mother	29.5 Years (SD ±5.7)	
Caregivers marital condition		
Living together	231	84.0%
Separated / Divorced/ single parents	44	16.0%
Religion of the household head		
Catholicism	83	30.2%
Protestant	131	47.6%
Others	61	22.2%
**Socio-demographic characteristics**		
Distance from home to their usual health facility		
Less than 30 min	109	39.6%
30 min to 1 hour	99	36.0%
> 1 hour	67	24.4%
Highest maternal educational level		
Secondary or higher educational level	117	42.5%
Primary education or less	158	57.5%
Number of siblings / other children at home		
None	67	24.4%
1 sibling	82	29.8%
More than 1 child /sibling	126	45.8%
Wealth index		
Rich (Ubudehe 3-4)	88	32.0%
Poor (Ubudehe 1-2)	187	68.0%
Type of insurance		
RSSB, MMI, Private	78	28.4%
No insurance	13	4.7%
Community Health Insurance (“Mutuelle”)	184	66.9%
Type of health facility		
Private health facility	59	21.4%
Public health facility	216	78.6%

**Socio-demographic and clinical/illness characteristics and relation to health seeking behaviour:** On bivariate analysis of socio-demographic factors, those significantly associated with delayed health seeking were female sex of the sick child (p = 0.05), having single parents (p = 0.015), higher wealth index (p = 0.006) and use of a public health facility (p = 0.003) (Table 2). Among all clinical characteristics, factors significantly positively associated with early health seeking were presence of convulsions (p = 0.007), parents perceiving the illness as severe (p < 0.001), no use of un-prescribed medicine (p < 0.001), no use of special prayers (p < 0.001), and no use of traditional healing (p < 0.001) (Table 3). On multivariate logistic regression analysis (Table 4), the significant predictors of delayed seeking care remained parents perceiving the illness as not severe (p < 0.001), use of unprescribed medicine (p = 0.044), use of special prayers before seeking care (p < 0.001), use of a public health facility (p = 0.004, and no use of traditional healing (p < 0.001). Traditional medicines were the second most common ′medication′ that was given by parents prior to presentation at a health facility, representing 44% of all the medications that were “self-prescribed”.

**Table 2:** Socio-demographic characteristics and relation to health seeking behaviour

Characteristics	Early seeking health care (n=178)	Delayed seeking health care (n=97)	Unadjusted Odd Ratio (OR) (95% C.I.)	P value
	Frequencies (%)	Frequencies (%)		
**Total number**	178 (64,7%)	97 (35, 3%)		
**Child's characteristics**				
Sex of the child				
Male	119 (71, 2%)	48 (28, 7%)	Ref	
Female	59 (54, 6%)	49 (45, 4%)	OR=2,05 (1,24-3,41)	p=0,005
**Caretaker's characteristics**				
Age of the mother				
≤ 20 years	7 (46, 6 %)	8 (53, 4 %)	Ref	
21 to 30 years	91 (65 %)	49 (35 %)	OR=0,47 (0,16-1,37)	p=0,169
>30years	80 (66, 6 %)	40 (33, 4 %)	OR=0,43 (0,15-1.30)	P=0,135
**Caregivers marital condition**				
Single parents	11 (42, 3 %)	15 (57, 7 %)	Ref	
Separated / Divorced	12 (66, 6 %)	6 (33, 4 %)	OR=0,36 (0,10-1,28)	p=0,116
Both parents living together	155 (67 %)	76 (33 %)	OR=0,36 (0,16- 0,82)	p=0,015
**Socio-demographic characteristics**				
Distance from home to their usual health facility				
Less than 30 min	78 (71, 5 %)	31 (28, 5 %)	Ref	
30 min to 1 hour	59 (59, 6 %)	40 (40, 4 %)	OR=1,70 (0,95- 3,04)	p=0,07
> 1 hour	41 (61, 2 %)	26 (38, 8 %)	OR=1,60 (0,84- 3,03)	p=0,155
**Educational level of the mother**				
No education	10 (55, 6 %)	8 (44, 4 %)	Ref	
Some educational level not greater than primary school	80 (57, 1 %)	60(42, 9 %)	OR=0,93 (0,35- 2,51)	p=0,898
Secondary or higher educational level	88 (75, 2 %)	29(24, 8 %)	OR=0,41 (0,15-1,14)	p=0,088
**Number of siblings / other children at home**				
No other	42 (62, 7 %)	25 (37, 3 %)	Ref	
1 sibling	56 (68, 3 %)	26 (31, 7 %)	OR=0,78 (0,39- 1,53)	p=0,473
More than 1 child /sibling	80 (63, 5 %)	46 (36, 5 %)	OR=0,96 (0,52- 1,78)	p=0,912
**Wealth index (New Ubudehe classification)**				
Lowest (Ubudehe 1-2)	108 (57, 7 %)	79 (42, 3 %)	Ref	
Highest (Ubudehe 3-4)	70 (79, 5 %)	18 (20, 5 %)	OR=0,35 (0,19-0,63)	p=0,006
Type of insurance				
No insurance	8 (61, 5 %)	5 (38, 5 %)	Ref	
Community Health Insurance (“Mutuelle”)	107 (58, 1 %)	77 (41, 9 %)	OR=1,15 (0,36-3,65)	p=0,811
Others	63 (80, 8 %)	15 (19, 2 %)	OR=0,40 (0,10-1,33)	p=0,131
**Type of health facility**				
Private health facility	48 (81, 3 %)	11 (18, 7 %)	Ref	
Public health facility	130 (60, 2 %)	86 (39, 8 %)	OR=2,88 (1,42-5,87)	p=0,003

**Table 3:** Clinical or illness characteristics related to health seeking

Characteristics	Early seeking health care(n=178)	Delayed seeking health care (n=97)	Unadjusted Odds Ratio (OR) (95% CI)	P value
	Frequencies (%)	Frequencies (%)		
**Total number**	178 (64, 7%)	97 (35, 3%)		
**Traditional healers intervention**				
No	175 (72, 3 %)	67 (27, 7 %)	Ref	
Yes	3 (9 %)	30 (91 %)	OR=26,12 (7,71-88,45)	p<0,001
**Caretaker perception about severity of illness**				
Severe	151 (79, 5 %)	39 (20, 5 %)	Ref	
Moderate or mild	27 (31, 7 %)	58 (68, 3 %)	OR=8,32 (4,67-14,80)	p<0,001
**Use of special prayers before consulting**				
No	169 (70, 7 %))	70 (29, 3 %)	Ref	
Yes	9 (25 %)	27 (75 %)	OR=7,24 (3,24-16,19)	p<0,001
**Use of unprescribed medicine at home**				
No	119 (78, 2 %)	33 (21, 8 %)	Ref	
Yes	59 (47, 9 %)	64 (52, 1 %)	OR=3,91 (2,32-6,60)	p<0,001
**Presence of convulsions**			Ref	
Yes	38 (82, 6 %)	8 (17, 4 %)	OR=0,95 (0,57-1,60)	p=0,859
No	140 (61, 1 %)	89 (38, 9 %)		
**Mortality**			Ref	
No	175 (65, 3 %)	93 (34, 7 %)	0,95 (0,27-3,33)	p=0,859
Yes	3 (42, 8 %)	4(57, 2 %)		
**Presence of fever**			Ref	
Yes	110 (64, 3 %)	61 (35, 7 %)	OR=0,73 (0,28-1,89)	p=0,235
No	68 (65, 4 %)	36 (34, 6 %)		
**CPR and/or BMV in the first 24 h of admission**			OR=26,12 (7,71-88,45)	p<0,001
Yes	7 (63, 6 %)	4 (36, 4 %)		
No	171 (64, 8 %)	93(35, 2 %)	Ref	
**Admission to PICU in the first 24h**			OR=8,32 (4,67-14,80)	p<0,001
Yes	11 (57, 9 %)	8 (42, 1 %)		
No	167 (65, 2 %)	89 (34, 8 %)	Ref	

BMV=Bag-Mask Valve ventilation; PICU=Pediatric Intensive Care Unit; CI=Confidence interval

**Table 4:** Multivariate logistic regression analysis of categories of perceived predictors as determinants of delay in seeking health care for more than 48 hours

Characteristics	Adjusted Odds Ratio (AOR) (95% CI))	P values
**Traditional healers intervention**		
No	Ref	
Yes	AOR=13.27 (3.42, 51.49)	p<0.001
**Caretaker perception about severity of illness**		
Severe	Ref	
Not severe	AOR=8.20 (4.08, 16.47)	p<0.001
**Use of special prayers before consulting**		
No	Ref	
Yes	AOR=6.42 (2.50, 16.48)	p<0.001
**Type of Health facility consulted**		
Private health facility	Ref	
Public hospital	AOR=4.00 (1.54, 10.39)	p=0.004
**Use of unprescribed medicine at home**		
No	Ref	
Yes	AOR=2.00 (1.01, 3.91)	p=0.044

Ref = Reference group; CI = Confidence Interval

**Morbidiy:** Morbidity was defined in term of admissions to PICU in the first 24 hours (p=0.235) and CPR and/or BMV done within the first 24 hours of admission in the hospital (p=0.859). These were not found to be associated with a delayed healthcare seeking (Table 3).

**Mortality:** Mortality was not associated with delayed presentation. Though not statistically significant there was a trend towards increased mortality in the delayed presentation group with and odds ratio (OR) of 2.51 (95% CI = 0.55 to 11.451, p = 0.24) (Table 3).

## Discussion

This research project aimed to answer the question “How prevalent is delay in seeking healthcare, what are its predictors and its immediate outcome in term of morbidity and mortality among children, under 5-years-of-age, in Rwanda” Thirty-five percent of children presenting to a pediatric ED unit at a tertiary hospital sought care after 48 hours of the illness onset. The most significant predictors of delay in seeking care were use of traditional healers (AOR = 14.87, 95% CI: 3.94-56.12), the recognition of illness as mild (AOR = 8.20, 95% CI: 4.08-16.47), use of un-prescribed medicine at home (AOR = 2.00, 95% CI: 1.01-3.91), use of special prayers provided by ministers of God before seeking healthcare (AOR = 6.42, 95% CI: 2.50, 16.48), and first consultation at public institutions (AOR = 4.00, 95% CI:1.54-10.39). Twelve percent of all interviewed caregivers sought the intervention of traditional healers before seeking any help at a formal health facility. This proportion is very high compared to the number reported in a study done in Kenya in which the prevalence of first seek the intervention of a traditional healer for the sick child was 6.3% [22]. Multiple studies done in developing countries have reported that the intervention of traditional healers is an important barrier in health care-seeking behavior [17,23-33]. It has been reported that the main reasons reported by caregivers for seeking the intervention of traditional healers were that tradiational healers were perceived as being able to determine the fate and outcome of any illness [34], and to deal with all spiritually related illness and witchcraft [35,36]. Many caregivers have been shown to think that some diseases were not for medical treatment [37]. After controlling for confounders in multivariate analysis, the use of special prayers before seeking care was the third most important factor associated with delayed care seeking. Those prayers were delivered by either specialized men of prayers or the servants of God (known as “men of God”), or both.In a qualitative study done in Kenya, Niger and Nigeria, religious beliefs were found to be an important predictor of the care-seeking behavior and the authors discussed that in some area, some denominations prohibited care-seeking at the level of health facilities [10].

Another notable finding in our study was the use of un-prescribed medication (self-medication), which was strongly associated with a delayed care-seeking behavior. Those findings are supported by other studies done in many countries [38,39] in which the self-medication with over-the counter drugs or other drugs was shown to interfere with early and accurate care-seeking at a formal health facility. The use of un-prescribed medication is challenging as many illnesses are simple and can benefit from simple, un-prescribed, medications, given at home. Therefore more in-depth work needs to be done to identify positiveand negative uses of self-medication. Regulation of over-counter medication is also important so that parents are “required” to seek medical attention in order to gain medical attention. Early mortality was a secondary outcome in our study. We identified a trend towards increased mortality in the delayed presentation group with and odds ratio (OR) of 2.51 (95% CI = 0.55 to 11.451, p = 0.24). However, our study was not powered to statistically identify an association and the sample size was not sufficient to be able to show any statistical significance in relationship to mortality. Previous research has demonstrated that delaying care seeking and/or poor case management contributes to increased mortality [40,41] and our trend would suggest a similar finding. In the experience of the authors the overall mortality rate found in this sample does not reflect the true rate from our clinical experience. This finding could reflect a period of low mortality or recruitment bias because of fear of recruiting after the death of a child or only recruiting children presenting during the day which may have missed children who died during the night hours.

This study is the first in our country focusing on which factors could be playing a role in the care-seeking behavior of caregivers. We recognize that our study had several limitations. We used an established definition of “delayed health seeking” of > 48 hours from illness onset. Clearly many children have minor illness and may not need any medical intervention. An alternative would have been to define delayed care in terms of late health seeking in the presence of illness requiring medical intervention. This would have made for an interesting analysis but would have added a significant subjective nature to definition. As a cross-sectional study, although an association was made between many factors and the outcome, causality cannot be established. The possibility that the caregivers could have forgotten or omitted some useful information (recall bias) cannot be excluded. Another limitation is linked to the generalizability of our results. This study has aimed to describe the prevalence of delayed health seeking. In doing so, it has answered the question in relation to a tertiary health setting. It is important to note that a significant number of pediatric deaths in sub-Saharan Africa occur in the community. Many of these caregivers will not seek care or will seek care from other sources, such as traditional healers and family members. These children have not been considered in our results and findings. The results here do not identify a higher mortality in late-presentation. But as discussed these could reflect under powering of the study because of a low overall mortality rate. It is therefore difficult to know how policy makers should respond to the results. If there is no increased mortality should steps be taken? Irrespective, it is worth noting that negative practices by parents (traditional medicine, praying for a child, self-medicating) continue to be present and affect health seeking practice. Though they may be seen as “adjuncts” to medical care they should not delay care. Education and poverty eradication would appear the obvious responses, though this is not based on the results of this study.

## Conclusion

Rwanda has sustained tremendous achievements in strengthening the community-based health systems. Our study showed that pre-hospital Caregivers' delay in health-care seeking for sick under-five children admitted in a pediatric ED is a common and multifactorial problem. As interdependency between those factors might exist, further studies at the community level are needed to fully assess and measure their respective role in health-care seeking behavior.

### What is known about this topic

- Caregiver delay in seeking healthcare is multifactorial;
- Delayed care seeking practice has been found to be associated with increased morbidity and mortality in pediatric population.

### What this study adds

- This study highlights that consulting a traditional healer is the most strongly associated with caregiver delay in seeking healthcare among children seeking a primary health facility in Rwanda;
- This study was underpowered to identify any association between delay in seeking healthcare and mortality.

## Competing interests

The authors declare no competing interest.
